# Implementation of a virtual, shared medical appointment program that focuses on food as medicine principles in a population with obesity: the SLIM program

**DOI:** 10.3389/fnut.2024.1338727

**Published:** 2024-06-19

**Authors:** Kyleigh Kirbach, Imani Marshall-Moreno, Alice Shen, Curtis Cullen, Shravya Sanigepalli, Alejandra Bobadilla, Lauray MacElhern, Eduardo Grunvald, Gene Kallenberg, Maíra Tristão Parra, Deepa Sannidhi

**Affiliations:** ^1^School of Medicine, University of California San Diego, San Diego, CA, United States; ^2^University of California San Diego Health, San Diego, CA, United States; ^3^Herbert Wertheim School of Public Health and Human Longevity Science, University of California San Diego, San Diego, CA, United States; ^4^Center for Integrative Medicine, University of California San Diego, San Diego, CA, United States; ^5^Bariatric and Metabolic Institute, University of California San Diego, San Diego, CA, United States; ^6^International Consulting Associates, Inc., Arlington, VA, United States; ^7^Department of Family Medicine, University of California San Diego, San Diego, CA, United States

**Keywords:** shared medical appointment, lifestyle medicine, food as medicine, implementation science, obesity

## Abstract

**Background:**

Multimodal lifestyle interventions, employing food as medicine, stand as the recommended first-line treatment for obesity. The Shared Medical Appointment (SMA) model, where a physician conducts educational sessions with a group of patients sharing a common diagnosis, offers an avenue for delivery of comprehensive obesity care within clinical settings. SMAs, however, are not without implementation challenges. We aim to detail our experience with three implementation models in launching a virtual integrative health SMA for weight management.

**Methods:**

Eligible patients included individuals 18 years of age or older, having a body mass index (BMI) of 30 kg/m^2^ or 27 kg/m^2^ or greater with at least one weight related comorbidity. The Practical, Robust Implementation and Sustainability Model (PRISM), Plan, Do, Study, Act (PDSA), and the Framework for Reporting Adaptations and Modifications-Enhanced (FRAME) models were applied to guide the implementation of the Supervised Lifestyle Integrative Medicine (SLIM) program, a virtually delivered, lifestyle medicine focused SMA program, in a weight management clinic within a major health system. We describe how these models, along with attendance for the initial cohorts, were used for decision-making in the process of optimizing the program.

**Results:**

172 patients completed the SLIM program over two years. Attendance was lowest for sessions held at 8:00 AM and 4:00 PM compared to sessions at 10:00 AM, 1:00 PM, and 3:00 PM, leading to only offering midday sessions (*p* = 0.032). Attendance data along with feedback from patients, facilitators, and administrative partners led to changes in the curriculum, session number and frequency, session reminder format, and intake visit number.

**Conclusion:**

The use of implementation and quality improvement models provided crucial insight for deployment and optimization of a virtual, lifestyle medicine focused SMA program for weight management within a large healthcare system.

## Introduction

Obesity has become a global epidemic, with two billion people currently living with excess weight and projected estimates that more than half of the world’s population having overweight or obesity by 2035, according to the World Obesity Atlas (1). In response to increasing evidence suggesting that lifestyle modifications are effective in treating obesity, the U.S. Preventive Services Task Force is currently recommending intensive, multimodal lifestyle programs that focus on effective dietary strategies, physical activity, and behavior change as the first-line treatment of obesity (2).

The Diabetes Prevention Program (DPP) demonstrated that intensive lifestyle intervention (ILI) was effective for weight loss and preventing or delaying the onset of type 2 diabetes. In its randomized design, participants in the ILI treatment group lost significantly more weight than those in the metformin only and placebo groups (3). Notably, the ILI featured in the DPP utilized the “Food as Medicine” paradigm to treat and reverse disease, establishing a healthy dietary pattern as a foundation for treatment. By strategically using food as a therapeutic tool and making deliberate choices about the types and quantities of foods consumed, individuals with obesity can achieve sustainable weight loss (4–6), reduce the risk of obesity-related comorbidities (7, 8), and improve their overall quality of life (9).

While these results are promising, using the Food as Medicine paradigm in primary care or clinical settings is challenging due to the limited time during usual appointments dedicated that is inadequate for intensive lifestyle counseling. The most common format of healthcare delivery involves a brief 15-to-20-min visit for an acute complaint, management of chronic disease or wellness checkup, which may include basic lifestyle counseling but is typically not enough time to develop a cohesive plan for weight management (10). The adjunctive use of Shared Medical Appointments (SMAs) can successfully address this issue by providing patients with cost-effective appointments dedicated to intensive lifestyle counseling and weight management planning (11). These medical appointments, facilitated by a physician, involve a small group of patients with a common diagnosis and can last 60-to-120-min. Patients receive clinical care such as check-ins with a physician, physical exams, medication management, patient education, and peer support (12, 13). SMAs have been shown to improve patient access, self-efficacy, self-care (13), clinician and patient satisfaction (14), and clinical outcomes (11, 15), while reducing cost (15) and repetition of health information by the practitioner due to the group session format (16). Notably, patients with obesity who attend weight management SMAs have been found to sustain significantly more weight loss for up to 12 months compared to patients who receive the standard of care (17).

Despite the advantages of SMAs, many clinics still do not offer them, possibly due to implementation barriers such as resource allocation, scheduling challenges, and lack of staff familiarity with associated billing and reimbursement procedures (12, 18, 19). Difficulty with patient recruitment and low program adherence have also been barriers to program sustainability (20). The lengthy, multi-session format of most SMA programs has also elicited patient feedback about scheduling conflicts, hardship in obtaining transportation and childcare, and complaints about extended visit lengths (21). Even with reports of numerous challenges in implementation, few feasibility papers have examined the process of program implementation and adaptation (22), which may provide valuable insight for clinics interested in establishing SMA programs of their own (16).

The 2013 AHA/ACC/TOS Guideline for the Management of Overweight and Obesity in Adults call for additional research in “effective methods of delivering lifestyle interventions remotely” (23). Virtual delivery of SMA programs may reduce overhead costs and improve treatment reach by minimizing patient barriers such as access to transportation, distance to the clinic, and access to childcare (21) but may prove challenging in other ways like access to a stable internet connection and use of web-based video conferencing (24).

The Supervised Lifestyle Integrative Medicine (SLIM) program is a virtual, lifestyle medicine-focused SMA program delivered as an adjunct to traditional clinic-based obesity treatment. We describe our use of the Practical, Robust Implementation and Sustainability Model (PRISM) implementation model and the Plan, Do, Study, Act (PDSA) and Framework for Reporting Adaptations and Modifications-Enhanced (FRAME) quality improvement models to implement and adapt the SLIM program to be acceptable and sustainable within a large healthcare system.

## Methods

### Patients and setting

Patients were referred to the SLIM program by physicians at the University of California, San Diego (UCSD) Center for Advanced Weight Management. We received support from the Center for Integrative Health, the UCSD Department of Family Medicine, and the UCSD Bariatric and Metabolic Institute early on to ensure a sustainable home for the program and a referral pipeline. Upon referral, eligibility was confirmed independently via medical record review by the research team and the patient navigator, who also interviewed patients individually. The inclusion criteria were being 18 years of age or older, having a body mass index (BMI) of 27 kg/m^2^ or greater with weight-related comorbidity, or a BMI of 30 kg/m^2^ or greater. Participation in the program was determined based on insurance eligibility. During enrollment, patients were informed that they would receive medical care in a group setting and that the program would be delivered virtually through Zoom video conferencing. The patient navigator received verbal confirmation that the patient had stable internet access, a device to access the visits, and were comfortable using the required technology. Patients signed an agreement that medical information disclosed by others during visits would not be shared outside of the medical visits. An exemption was granted by the University of California Human Research Protections Program for the retrospective collection of attendance and demographic data from the electronic medical record.

### SLIM program intervention

The SLIM program is an intensive weight management intervention developed at UCSD. It comprises a series of virtual, SMAs to deliver a Lifestyle Medicine-based curriculum to patients to improve their weight management and health outcomes. The curriculum was developed following the key tenets of Lifestyle Medicine: whole-food, plant-predominant diet, regular physical activity, restorative sleep, stress management, reduction of risky behaviors, and positive social connections. There was an intentional focus on leveraging healthy eating patterns to improve patients’ metabolic health profile and was intended to be delivered as an adjunct to traditional clinic-based obesity treatment.

Each curriculum session was approximately 1–2 h in length and covered various topics in lifestyle medicine, including but not limited to nutrition, culinary medicine, exercise, sleep, and stress. The curriculum format for each session was structured with the following components: welcome/ice breaker activity and introductions, educational session on a lifestyle medicine-related topic (Table 1), discussion, questions and answers, and ending with medication adjustments or any other specific activity that was needed to support patients. Sessions included speaker lectures, group discussions, and other group exercises to create an interactive, two-way learning environment. To foster trust and connection among both patients and facilitators, each session began with a reminder that the SMA was considered a “safe space” for self-disclosure and a statement that all information shared would remain strictly confidential within the group session.

**Table 1:** Initial and updated SLIM curriculum.

Session	Initial	Updated
1	SMART goal setting	Welcome to SLIM
2	5 pillars of a healthy diet	SMART goal setting
3	Overcoming triggers	5 pillars of a healthy diet
4	Sleep hygiene	Principles of positive psychology and strengths-based thinking, self-compassion
5	Environmental influences	Cognitive Behavioral Therapy (CBT), applying CTFAR, environmental influences
6	Stress management	Hunger and snacking, sugars, intuitive eating, mindfulness
7	Grocery shopping, whole grains	Physical activity
8	Protein, fat, sodium	Meal prepping, grocery shopping, food labels
9	Meal prepping	Calorie density, types of carbohydrates
10	Strengths-based thinking	Sleep hygiene, stress management, social connection
11	Gratitude	Relapse prevention
12	Hunger and snacking, sugars	Gratitude
13	Physical activity	
14	Leisure time, reducing sedentary behavior	
15	Social support	
16	Sustainability and weight reduction maintenance	

Food as medicine-based topics comprised more than a third of the curriculum content. They included dedicated sessions on fundamental topics such as caloric density, nutrition, and the benefits of incorporating legumes, whole grains, and seasonal produce into everyday diets. Although plant-based diets were strongly emphasized, patients were introduced to various other balanced diets, including MyPlate (25), DASH (26), and Mediterranean diets (27, 28), and advised to select one that would be most appropriate for their socio-cultural background. Patients were encouraged to abandon “fad”-based thinking about dieting and reframe their understanding of nutrition in a holistic fashion that would be sustainable for long-term dietary change. The curriculum also emphasized “real-world” applications of culinary medicine in a dedicated session about grocery shopping strategies, understanding nutritional labels, meal planning, preparation, and batch cooking. Recipe and meal sharing were strongly encouraged throughout group discussions and other “show-and-tell”-based activities.

Behavioral change techniques and mental health-related topics, including mindfulness, mindful self-compassion, and cognitive behavioral therapy (CBT) were fundamental and interwoven throughout every topic in the SLIM curriculum. Each session began with a guided mindful meditation session followed by a “Rose, Bud, Thorn” activity to provide patients with time to reflect upon and share their stories of success, challenges, and opportunities for growth since the prior session. Behavioral change techniques (29) like Specific, Measurable, Achievable, Realistic, and Timely (SMART) goal setting (29) for initiating and maintaining change were repeatedly emphasized and practiced throughout each session. Cognitive behavioral therapy-based techniques and models were also used in various sessions to contextualize negative thoughts and feelings about lifestyle change and guide patients in overcoming psychological barriers to sustaining their lifestyle goals. The curriculum also included an entire session about relapse prevention (29) with an emphasis on resilience, problem solving, self-care, and self-compassion. Full-length curriculum materials and details are freely available online on our program website.^1^

### Initial implementation design

Upon enrollment into the SLIM program, patients received one, 60-min, one-on-one intake visit with an attending physician certified in Obesity Medicine and Lifestyle Medicine. Patients were introduced to the program, a lifestyle and weight history was taken, and baseline anthropometric and laboratory studies were performed including routine vitals, height, weight, waist circumference, complete metabolic panel (CMP), fasting insulin, lipids panel, C-reactive protein (CRP) and thyroid-stimulating hormone (TSH). If indicated, anti-obesity medications (AOMs) were also initiated at this time. The primary goal of the intake visits was to establish rapport with the patients, understand any psychosocial factors affecting their lifestyle, and determine readiness and barriers to change.

Following the intake visits, patients were assigned to a cohort and provided instructions to attend virtual SMAs via group Zoom calls. The original program began with 1 session each week for four sessions, then moved to 1 session every two weeks for 4 sessions (spanning 8 weeks or roughly 2 months), then 1 session every three weeks for 4 sessions (spanning 12 weeks or roughly 3 months), and finally 1 session monthly for 4 sessions (spanning four months). In total, the 16 sessions were completed sequentially and occurred over about 10 months. The SMAs were led by the same attending physician alongside a team comprised of a health coach and Preventive Medicine resident physicians.

After completing the 16 SMAs, patients were seen by the attending physician for two more follow-up in-person visits for laboratory studies, as well as a discussion of maintenance plans and future goals. Patients were encouraged to continue following up with the same physician and incorporating healthy lifestyle habits through referrals to community events, such as free cooking classes and group walks.

### Implementation design and adaptation

#### PRISM-guided implementation design

**Figure 1:**
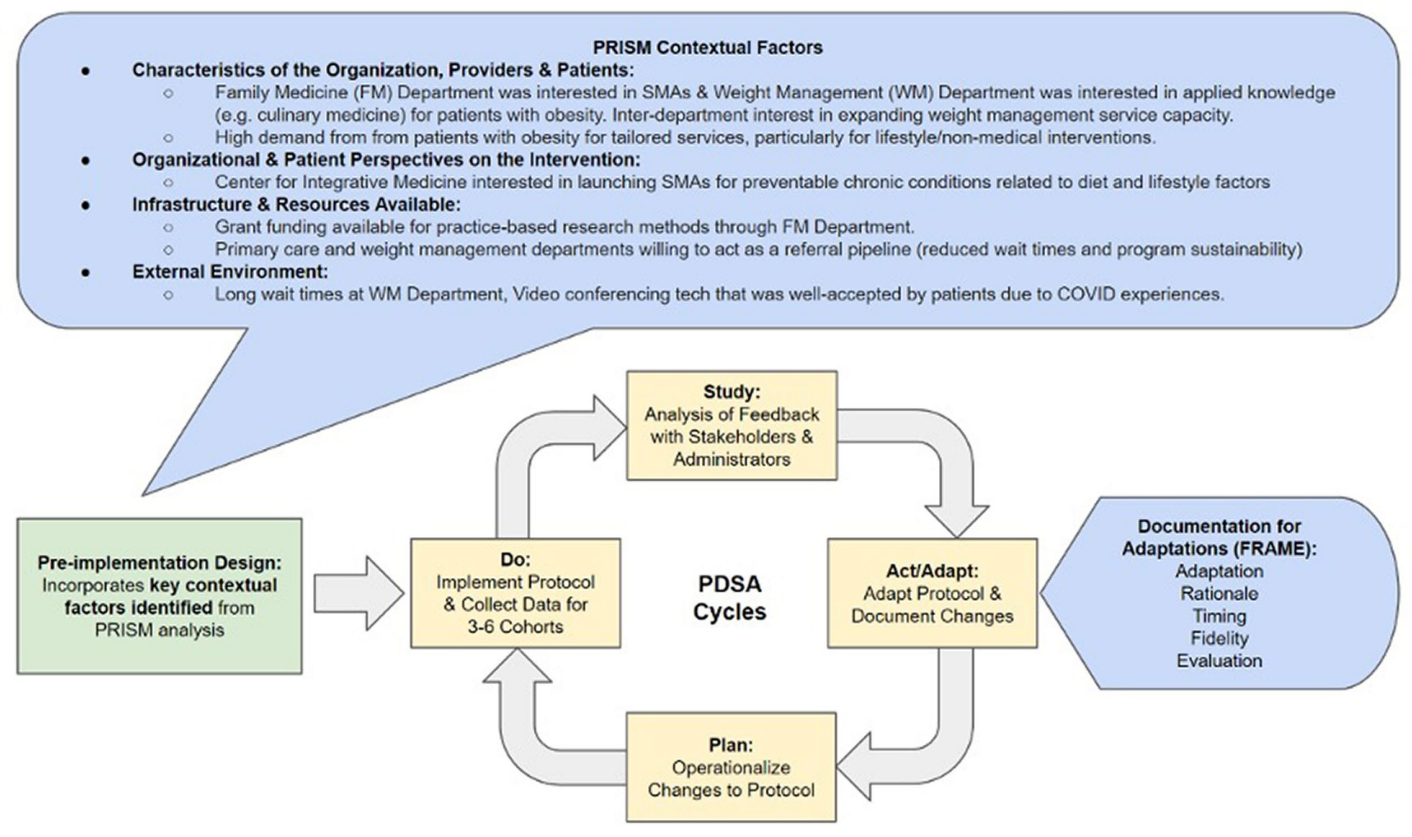
Application of implementation science frameworks.

We used multiple implementation science frameworks to create a structure to guide our implementation, evaluation, and adaptation of the SLIM program within the context of our organizational setting and patient characteristics. We used the Practical, Robust Implementation and Sustainability Model (PRISM) to inform our implementation approach, which is comprised of four key domains: (1) organizational and participant characteristics (patients and providers); (2) characteristics of the intervention from the organizational and participants’ perspectives; (3) availability and sustainability of infrastructure/resources, and (4) the external environment and context (30). Our usage of the PRISM model and the key findings from each domain is presented in Figure 1.

#### PDSA-driven program adaptation with FRAME

Following the implementation design process using the PRISM framework, the SLIM program was implemented in rolling cohorts over three years. The Plan-Do-Study-Act (PDSA) model was used iteratively to adapt the implementation plan throughout active program administration. Adaptations made during the ‘Study-Act’ phases of PDSA were documented using parameters drawn from the Framework for Reporting Adaptations and Modifications-Enhanced (FRAME) model (31).

The PDSA model conceptualizes program testing cycles as a process of developing a plan to execute an implementation (Plan), carrying out the plan while gathering data (Do), analyzing the outcome data (Study), and using the data to adapt the next iteration of the implementation (Act) (32). Our PDSA cycles were operationalized through administration of the program for a span of three to six cohorts while conducting periodic research group meetings for review of feedback and outcome data collected. During these meetings, decisions were made to adapt the implementation plan on an ongoing basis using outcome data, team consensus, and input from patients, clinic staff, and administrative partners.

Adaptation decisions were systematically analyzed using FRAME to create structured and meaningful documentation along six domains – the adapted component, the nature of the adaptations, rationale, timing, fidelity, and post-adaptation evaluation. Using a structured documentation framework facilitated internal consistency in quantifying and qualifying the impact of our adaptations on implementation outcomes. A complete overview of the usage of implementation science (IS) frameworks to design and implement the SLIM program is outlined in Figure 1.

### Outcome measures and data analysis

Qualitative data informally collected from the implementation included feedback from key partners (e.g., health coaches, patients, clinic staff, and directors) and patients. Data was collected using a combination of *ad hoc* conversations and feedback sessions following cohort cycles, during which feedback regarding the patients’ satisfaction with the SMAs and the lifestyle medicine interventions was actively solicited. Input from key partners and patients guided adaptations to the program design and implementation.

Quantitative attendance data for SLIM patients who participated during year one and year two was collected from the UCSD Epic electronic medical record database to analyze attendance stratified by sessions and cohorts. For the quantitative analysis, statistical methods were performed with IBM SPSS Standard v29 to examine the effect of varying session days and times on patient attendance. Session-level attendance was defined as the number of attendees present as a percent of the number of patients scheduled. The correlation between session-level attendance and program adaptations was assessed using an independent samples *t*-test. For a power level of 90%, at least 56 patients need to be in each comparison group.

## Results

### Patient demographics

172 patients completed the SLIM program over three years (age: 51.2 ± 14.6 years; 76% female, 23% male, 1% nonbinary) (Table 2). 4 cohorts finished in year 1, 8 in year 2, and 11 in year 3, with cohort sizes reaching a maximum of 17 patients. Out of those who completed the program, baseline metabolic data was complete for 130 patients: BMI was 37.2 ± 7.5, kg/m^2^, LDL cholesterol was 107 ± 33 mg/dL, triglycerides were 129 ± 63 mg/dL, and HbA1c was 5.6 ± 0.6%.

**Table 2:** Baseline demographic and metabolic variables.

Baseline demographic information	*n* = 172
Mean age, y (SD)	51.2 (14.6)
Sex, *n* (%)	
Female	130 (76)
Male	40 (23)
Nonbinary	2 (1)
Race, *n* (%)	
American Indian/Alaska Native	1 (1)
Asian	11 (6)
Black or African American	23 (13)
Native Hawaiian or other Pacific Islander	1 (1)
Other or Mixed Race	35 (20)
White	99 (58)
Unknown/Not Reported	2 (1)
Ethnicity, *n* (%)	
Hispanic or Latino	31 (18)
Not Hispanic or Latino	103 (60)
Unknown/Not Reported	38 (22)
Baseline metabolic data	*n* = 130
BMI, kg/m^2^ (SD)	37.2 (7.5)
Lipids	
LDL, mg/dL (SD)	107 (33)
Triglycerides, mg/dL (SD)	129 (63)
Glucose, mg/dL (SD)	100 (15)
HbA1c, % (SD)	5.6 (0.6)

### Attendance

Attendance data for years 1 and 2 are included in this analysis as year 3 was ongoing at the time of analysis (Table 3). The average attendance rate was lowest for cohorts 10 and 11, which had session times at 8:00 AM and 4:00 PM, respectively. The independent samples *t*-test revealed, among this sample of 199 sessions held during years 1 and 2, that sessions held at 8:00 AM or 4:00 PM had statistically significantly lower attendance rates (*M* = 0.42) than those who attended other sessions (*M* = 0.49) [*t*_(197)_ = −1.867, *p* = 0.032].

**Table 3:** Average attendance rate, year of program, day and time for each cohort (*n* = 199).

Program year	Cohort	Weekday or Weekend	Time	Average Attendance Rate	*n*
1	1	Weekday	8 AM	68%	11
	2	Weekday	10 AM	63%	12
	3	Weekday	1 PM	53%	13
	4	Weekday	1 PM	57%	14
2	5	Weekend	10 AM	57%	17
	6	Weekend	1 PM	33%	18
	7	Weekday	1 PM	36%	15
	8	Weekday	3 PM	60%	16
	9	Weekday	4 PM	37%	15
	10	Weekday	8 AM	32%	10
	11	Weekday	4 PM	29%	17
	12	Weekend	10 AM	40%	15

### Program adaptations

Table 4 shows the application of the Framework for Reporting Adaptations and Modifications-Enhanced (FRAME) model, which was used to provide a structured overview of adaptations made to the SLIM program during the first three years of program implementation. A combination of qualitative and quantitative data prompted adaptations to program implementation and content in an effort to meet the needs of patients, health coaches, clinic staff, physicians, and department leaders. Modifications made during year 2 included encouraging patients to communicate outside of the SLIM program session times as a source of social support (29) and refinement of the didactic material shared in group sessions to emphasize self-talk (29), self-belief, and a positive relationship with physical activity. Year 3 modifications centered around program execution with goals of improving attendance and clinic workflow.

**Table 4:** Program adaptations using the FRAME model.

Adaptation	Description	When?	Program year	Whose need was addressed?	Why was the adaptation made?
Encourage patient communication outside of the program sooner	Rather than waiting until the end of the program to encourage patients to connect with one another outside of the program, patients are now prompted during session 3	2023	3	Patients	Early establishment of an accountability partner and support system to encourage lasting lifestyle change
Change program structure	The program structure changed from 4 sessions weekly, 4 sessions every 2 weeks, 4 sessions every 3 weeks, and 4 sessions monthly to 12 sessions weekly.	2022	3	Patients, Department Administration	Improve attendance
Changing reminder method	No longer using email as a reminder method, relying primarily on MyChart messages, text messages and phone calls	2022	3	Patients, Health Coach/Facilitator	Improve attendance and participation, improve workflow efficiency
Changing session times	Removal of 8 a.m. and 4 p.m. sessions in response to attendance data providing insight into optimal session times (see Table 3)	2022	3	Health Coach/Facilitator	Improve attendance and participation, choose times with better attendance
Modified intake visit	Changed intake from 1, hour-long visit to 3, 30-min visits	2022	3	Physician, Department Administration,	If a patient cancels, only one appointment slot is lost; this gives patients other opportunities to meet with a physician before starting the program and enhances recruitment
Expansion of relapse sessions	Information added about Lapse vs. Relapse, addition of slides involving addiction and movement toward recovery	2021	2	Patients, Health Coach/Facilitator	Improve mental health and motivation
Adding information on weight stigma	Information added to better address the weight stigma, to improve patient/participant self-compassion	2021	2	Patients	Improve motivation, Improve patient self-love
Leisure activity moved ahead of physical activity	Leisure activity moved before physical activity to highlight enjoyable forms of movement	2021	2	Patients	Improve fit of content to patient/participant goals in response to a desire for more and earlier physical activity information
Added positive psychology	Added positive psychology pillars like the PERMA (Positive emotion, Engagement, Relationships, Meaning, Accomplishments) model of wellbeing	2021	2	Patients, Health Coach/Facilitator	Encourage focus on connection, gratitude, and living a meaningful life
Moved session on strengths	Moved the session on Strengths earlier in the curriculum	2021	2	Patients, Health Coach/Facilitator	Encourage strengths-based thinking at the beginning of the program
Recommend patients communicate outside of the program	Assigning patient team leaders and maintaining communication through Text/WhatsApp, an effort to improve patient support through communication	2021	2	Patients	Improve attendance and participation; improve mental health and motivation; improve program comradery

### Updated SLIM program

The SLIM program was updated after 12 cohorts of participants over two years of PDSA development cycles. Currently, upon enrollment into the program, participants are scheduled for 3, one-on-one intake visits with a physician certified in Obesity Medicine and Lifestyle Medicine. During these 30-min-long visits, patients are introduced to the program, a complete lifestyle and weight history is taken, and baseline laboratory studies are established. If indicated, AOMs are also initiated at this time. Following intake, participants are assigned to a cohort and provided instructions to attend 12 weekly virtual SMAs via group Zoom calls. The SMAs are led by the same intake physician alongside a team variably composed of a health coach, registered dietitian, and/or Preventive Medicine resident physicians. Each session is approximately 120 min in length, and the session topics covered a wide range of patient education on diet, culinary medicine, exercise, sleep, stress, and behavior change techniques, including mindfulness, mindful self-compassion, positive psychology, and cognitive behavioral therapy (CBT). Each session includes speaker lectures, group discussions, breakout groups, and other learning exercises. Full-length curriculum materials and details are freely available online on our program website (see Footnote 1). Following the 12 SMAs, patients are seen by the intake physician for two more follow-up visits for laboratory studies and discussion of maintenance plans and future goals. Comparison of the initial and updated SLIM curriculum can be found in Table 1.

## Discussion

The PRISM, PDSA, and FRAME models were employed to mitigate barriers and optimize implementation of a virtual, lifestyle medicine focused SMA program, providing an adaptive approach to patient-centered care. Use of the PRISM model during pre-implementation elucidated system and patient level barriers to program implementation and sustainability including departmental support, patient referral pipelines, and durable funding sources. Iterative rounds of the PDSA model led to identification of key factors influencing attendance and patient satisfaction such as optimal and nonoptimal session times, program structure and length, and education topics. Following identification of key factors, modifications based on these insights were systematically documented using the FRAME model.

We found session times of 8:00 AM and 4:00 PM had the lowest attendance. A possible explanation could be that these are common times when individuals start and end their workday making it likely they are in their work commute or actively working. Family needs such as transportation to school or to after school activities may also make those times challenging for individuals. Eisenstat et al. reports that in their experience, the optimal times of day for group medical visits depends on the population. For those working full-time, session times occurring before 8:30 AM and between 5:00 PM and 7:00 PM are best. For individuals who are retired, sessions between 12:00 PM and 2:30 PM are best (33).

Barriers to utilizing SMAs for chronic disease management in prior literature include inadequate administrative support and funding to develop and implement the program which impact scheduling and visit logistics and the establishment of a stable referral pipeline and, thus, a consistent revenue stream (22, 34). Walker et al.’s systematic review of SMAs for weight loss reported session time as a barrier to attendance, with sessions occurring during work hours attended the least. In addition, they point out that using implementation frameworks for planning and executing SMA programs are crucial, yet the use of such models in SMAs for weight loss have yet to be documented. Lastly, they report that the lack of early engagement with key partners as a limitation of prior studies of SMAs for weight loss (11). The use of implementation frameworks and quality improvement models such as PRISM, PDSA, and FRAME prevented many barriers to adoption seen in existing SMA literature. Identifying critical contextual factors and potential obstacles early through use of the PRISM model enabled us to address barriers to sustainability before implementation such as establishing departmental and administrative support, cultivating reliable patient referral sources, and securing the necessary resources and personnel. Executing regular cycles of the Plan-Do-Study-Act (PDSA) model allowed us to operationalize qualitative feedback from participants, health coaches, physicians, and staff and the quantitative data on attendance rates to optimize session times and refine the program structure, curriculum, and participant engagement strategies.

Limitations of this assessment include that the initial phase of the program was implemented in a real-world setting so there was not a control group and participants self-selected to enroll. Additionally, we were unable to collect all individual, social, and contextual variables influencing attendance. These variables may include but are not limited to socioeconomic status, geographical location, personal motivation, and unforeseen life events. Lastly, the virtual delivery of the SLIM program required that patients have access to the internet and videoconferencing technology which may be a financial or technological barrier, possibly leading to a patient population that is disproportionately from well-resourced areas and thus not representative of the general population.

Despite the limitations mentioned above, we believe the SLIM program is successful as its implementation has been sustained in the Center for Advanced Weight Management beyond its pilot phase and continues to grow in patient demand. The use of implementation models to guide its feasibility continues to be relevant and will actively guide the shape and future of the SLIM program. Moving forward, we envision the increased use of patient-centered outcomes like quality of life, mental health status, symptom management, and patient satisfaction to tailor adaptations. Future efforts include a study of effectiveness as well as continued evaluation of program attendance, scalability, and durability.

In conclusion, the use of implementation frameworks and quality improvement models provides valuable insights by offering a practical and multi-faceted approach to implementing and adapting a virtual, lifestyle medicine focused SMA program for weight management in a healthcare setting. Ongoing efforts are needed to enhance patient engagement and ensure long-term sustainability. If successfully implemented, and ultimate effectiveness is demonstrated, virtual SMA programs that offer comprehensive lifestyle education focusing on food as medicine can leverage the widespread adoption of virtual healthcare to make a meaningful contribution to the global efforts to reduce obesity.

## Data availability statement

The raw data supporting the conclusions of this article will be made available by the authors, without undue reservation.

## Ethics statement

The studies involving humans were approved by University of California, San Diego Human Research Protections Program. The studies were conducted in accordance with the local legislation and institutional requirements. Written informed consent for participation was not required from the participants or the participants’ legal guardians/next of kin in accordance with the national legislation and institutional requirements.

## Author contributions

KK: Writing – original draft, Writing – review & editing, Conceptualization, Data curation, Formal analysis, Methodology. IM-M: Data curation, Formal analysis, Writing – original draft, Writing – review & editing. AS: Data curation, Methodology, Writing – original draft, Writing – review & editing. CC: Data curation, Project administration, Writing – review & editing. SS: Methodology, Writing – original draft, Writing – review & editing. AB: Project administration, Writing – review & editing, Conceptualization, Investigation. LM: Conceptualization, Project administration, Resources, Writing – review & editing. EG: Conceptualization, Project administration, Resources, Writing – review & editing, Funding acquisition. GK: Conceptualization, Project administration, Resources, Writing – review & editing, Funding acquisition. MT: Conceptualization, Funding acquisition, Methodology, Project administration, Resources, Supervision, Writing – original draft, Writing – review & editing, Investigation. DS: Writing – original draft, Writing – review & editing, Funding acquisition, Investigation, Methodology, Project administration.

## Footnotes

^1^http://slim.ucsd.edu
